# Three-day delta-9-tetrahydrocannabinol (THC) exposure eliminates long-term depression in ventral tegmental area of young, but not adult mice

**DOI:** 10.1186/s42238-025-00287-7

**Published:** 2025-05-31

**Authors:** Michael Von Gunten, Seth Hoffman, Addison Smartt, Jeffrey G. Edwards

**Affiliations:** 1 Department of Cell Biology and Physiology, Brigham Young University, 4005 LSB, Provo, UT 84602 USA; 2 Neuroscience Center, Brigham Young University, Provo, UT 84602 USA

**Keywords:** Real-time quantitative PCR, QPCR, Cannabis, LTD, VTA, GABA, HDAC3, Plasticity, Adolescent, FAAH

## Abstract

Ventral tegmental area (VTA) dopamine signaling plays a key role in reward learning and drug dependence. VTA dopamine cell activity is regulated in part by local GABA interneurons, which participate in regulating reward prediction. Previously, our lab identified a cannabinoid type 1 receptor (CB1)-dependent form of excitatory long-term depression (LTD) in VTA GABA cells. LTD was eliminated in both young and adult mice after 7–10-day delta-9-tetrahydrocannabinol (THC) exposure. To build off these previous findings, we used mouse ex vivo brain slices to examine whether young mice undergo THC-induced alterations to VTA GABA cell plasticity after fewer exposures than their adult counterparts, as human adolescents have increased sensitivity to THC. Whole-cell electrophysiological recordings were performed on young (P14-P54) and adult (P66-P240) mice treated with THC or vehicle control for 3 days, after which we attempted to induce CB1-dependent LTD ex vivo. Plasticity was eliminated in young but not adult mice after 3 days of THC treatment. Because our previous work illustrated age-dependent alterations to mRNA transcripts after chronic THC-treatment, we also performed quantitative real-time PCR to assess any age dependent differences of 3-day THC exposure on mRNA levels in the VTA. Quantitative PCR revealed no THC-induced changes for young or adult mice but did show several differences between young and adult control mice. This age-dependent impact of THC on synaptic activity could reveal a physiological mechanism underlying increased sensitivity of adolescents to THC-induced alterations to plasticity.

## Introduction

Cannabis is the most prevalent illicit abused drug by human adolescents and young adults, with 10.5% of individuals aged 12 to 17 and 35.4% of people age 18 to 25 reporting cannabis use in 2021 (SAMHSA 2021). Not only are young individuals the largest consumers of cannabis, they are also at higher risk of negative impacts, such as impaired memory, decreased motivation, and social deficits (Dougherty et al. 2013; Volkow et al. 2014; Schaefer et al. 2021; Schaefer et al. 2023). Furthermore, those who use cannabis at a younger age are at a higher risk of developing cannabis use disorder (Millar, et al. 2021; Chen et al. 2005). Therefore, given that the likelihood of negative outcomes is higher during adolescence, and that these may persist through adulthood (Levine et al. 2017), it is crucial that we understand the biological mechanisms that contribute to this age-related susceptibility.

A key neural pathway for studying the effects of cannabis exposure is the mesolimbic system because it plays a role in drug dependence, reward learning and motivation (Schultz et al. 1997; Joffe et al. 2014). This pathway is comprised of the ventral tegmental area (VTA), which sends dopaminergic projections to the prefrontal cortex (PFC) and nucleus accumbens (NAc) (Koob and Volkow 2010). Exposure to drugs of abuse leads to long-term alterations to synaptic plasticity and gene transcription in these brain areas (Koob and Volkow 2010; Zehra et al. 2018). Given that endocannabinoid-mediated synaptic plasticity changes throughout development (Bernabeu et al. 2023) and can be altered after THC treatment (Mato et al. 2004; Friend et al. 2017), we aimed to investigate how modifications to endocannabinoid-mediated synaptic plasticity within this reward circuit contribute to age-related differences in response to THC-exposure. Here we examine THC-induced synaptic changes in the VTA of young and adult mice, identifying an inhibitory circuit modulated by cannabis exposure, which will allow us to parse out age-dependent differences in the role that cannabis-mediated synaptic modification plays in mesolimbic functioning.

Although much is known regarding the role that VTA DA cells play in drug dependence and reward learning, much less is known about the plasticity of VTA GABA cells that participate in regulating VTA DA cell activity. VTA GABA cells play a key role in reward consumption (Zessen et al. 2012), reward learning (Brown et al. 2012) and conditioned place preference (Bocklisch et al. 2013), but the effects that cannabis exposure has on these cells has not been fully explored. Previously, our lab characterized a novel cannabinoid type 1 receptor (CB1)-dependent form of long-term depression (LTD), at the excitatory inputs to VTA GABA cells (Friend et al. 2017). This plasticity has relevance to reward learning as it could lead to disinhibition of VTA DA neurons and thus mediate some of the rewarding effects of cannabis (Friend et al. 2017). We noted that chronic (7–10 days) THC treatment in vivo eliminates LTD and CB1 agonist-induced depression in both young and adult mice, but LTD and CB1 agonist-induced depression are still present after a single exposure to THC in both age groups (Friend et al. 2017; Ostlund et al. 2022). This is similar to findings from other labs, which demonstrate long-term THC exposure alters or abolishes endogenous plasticity in multiple brain areas (Hoffman et al. 2021). While both young and adult mice undergo THC-induced elimination of LTD after 7–10 days of treatment, it is unknown if young mice experience this elimination of plasticity after fewer exposures to cannabis compared to their adult counterparts. If young mice undergo THC-induced elimination of plasticity after fewer THC treatments, this may suggest greater sensitivity to THC-induced neuroadaptations in younger mice. This is important to investigate as cannabis use is especially negative for human adolescents, causing impaired cognition, abnormalities in the reward pathway, and increased probability of substance abuse (Volkow et al. 2014; Gilman et al. 2014; Hurd, et al. 2014). Thus, to follow up on our previously published work, we sought to determine if elimination of LTD in VTA GABA cells occurs after only 3 days of THC exposure in young and adult mice.

## Methods

### Animals

Male and female young (P14-P54) and adult (P66-P240) CD1 heterozygous GAD67-GFP knock-in mice produced by the Tamamaki laboratory (Tamamaki et al. 2003) that were bred in house and have been maintained for several years at our institution, were used to fluorescently identify VTA GABA cells. Our lab has used these mice to genetically identify VTA GABA cells previously (Friend et al. 2017; Nufer, et al. 2023) as only GABA cells express GAD67 in the VTA (Friend et al. 2017; Merrill et al. 2015; Chieng et al. 2011). Due to concerns of altered GABA transmission from tagging GAD67 with a GFP reporter, which could affect neurotransmission and plasticity, we performed experiments on wild-type littermate GABA cells in a past study (Bolneo et al. 2022). Utilizing spiking characteristics, post-hoc single-cell PCR, and absence of I_h_ current to identify GABA cells in slice, we noted no difference in plasticity characteristics between wild-type VTA GABA cells and VTA GABA cells tagged with GFP (Nufer, et al. 2023), and relevant to this study glutamate transmission/plasticity remained intact as well (Friend et al. 2017). Mice transition from juvenile to adolescent at approximately P28-35 and then again from adolescent to adult at approximately P60 based on puberty, epigenetic factors, and behavior (Laviola et al. 2003; Schneider 2013) therefore we considered all mice older than P60 to be adults, and all mice younger than P60 to be young mice. Because a clear transition between late adolescence and early adulthood is difficult to define, experiments were not performed on mice between P55-P65 to maintain a clearer boundary between age groups. No differences were noted within age groups; all adult mice and all young mice were kept within their groups for analysis.

### Drug treatments

THC pre-dissolved in EtOH was provided by the National Institute on Drug Abuse Drug Supply Program. Both young and adult mice were treated once daily for 3 days with an intraperitoneal injection (IP) of either THC (10 mg/kg, final injection volume of 100µL) or a vehicle control (EtOH in saline, final injection volume of 100µL). The vehicle control-treated mice were injected with the same volume to weight ratio of EtOH that was administered to the THC-treated mice. Injections were performed between 9 and 11 AM (4–6 h into the housing light cycle). Brain extractions for electrophysiology or quantitative real-time PCR (qRT-PCR) experiments were performed 24 h after the last injection.

### Slice preparation

Mice were anesthetized with isoflurane (1–2%) with respirations observed between 55–65 breaths/minute. Following anesthetization, we performed a cardiac perfusion of approximately 10 mL of oxygenated, ice cold NMDG-based cutting solution composed of 92 mM NMDG, 2.5 mM KCl, 1.25 mMNaH_2_PO_4_, 30 mM NaHCO_3_, 20 mM HEPES, 25 mM glucose, 2 mM thiourea, 5 mM Na-ascorbate, 3 mM Na-pyruvate, 0.5 mM CaCl_2_ dihydrate, and 10 mM MgSO_4_ heptahydrate (pH adjusted to 7.5 with HCl), after which they were decapitated with a rodent guillotine. Following the protocol designed by Ting et al. (Ting, et al. 2018) brains were rapidly removed and sectioned horizontally on a vibratome (250 μm) in oxygenated, ice-cold NMDG-based solution. After sectioning, slices were kept in a warm bath (34 degrees C) of the NMDG solution, and spiked with increasing amounts of NaCl (2 M) over 10 min after which they were placed in an oxygenated HEPES holding solution composed of 92 mM NaCl, 2.5 mM KCl, 1.25 mMNaH_2_PO_4_, 30 mM NaHCO_3_, 20 mM HEPES, 25 mM glucose, 2 mM thiourea, 5 mM Na-ascorbate, 3 mM Na-pyruvate, 2 mM CaCl_2_ dihydrate, and 2 mM MgSO_4_ heptahydrate (pH adjusted to 7.5 with 10 N NaOH).

### Electrophysiology

Mice were sacrificed between 9 and 11 AM (4–6 h into the housing light cycle). Recordings began at least one hour after brain extraction. Slices were placed in the recording chamber and bathed with oxygenated (95% O_2_, and 5% CO_2_) high divalent artificial cerebral spinal fluid (ACSF; 119 mM NaCl, 2.5 mM KCl, 1.15 mM NaH2PO4, 26 mM NaHCO3, 11 mM Glucose, 4 mM CaCl_2_ and 4 mM MgSO_4_) at 30–32 degrees Celsius. High-frequency stimulus (HFS; four, one-second 100 Hz bursts, each separated by 20 s) was the conditioning stimulus used to induce plasticity. Inhibitory GABAergic currents were blocked using 0.1 mM picrotoxin (ThermoFisher). The VTA was visualized using an Olympus BX51 WI microscope with a 40X water-immersion objective. GAD67-GFP-positive cells were selected for recording from the VTA at approximately the following coordinates from adult mouse bregma; anteroposterior − 3.2 to 3.3, mediolateral ± 0.6 to 0.7, dorsoventral − 4.5 to 4.3. Patch pipette resistance was 2.5–5.5 mΩ. Cells were held at − 65 mV and patched with a glass pipette filled with internal solution composed of 117 mm cesium gluconate, 2.8 mm NaCl, 20 mm HEPES, 5 mm MgCl_2_, 2.17 mM ATP-Na, 0.32 mM GTP-Na and 1 mm QX-314 (Tocris Bioscience), pH 7.28 (275–285 mOsm). Current traces were recorded using Multiclamp 700B amplifier (Molecular Devices). Signals were filtered at 4 kHz and digitized with an Axon 1550 A digitizer (Molecular Devices) connected to a Dell personal computer with pClamp 10.7 Clampex software (Molecular Devices).

### qRT-PCR

Under a dissecting microscope, the VTA region was located within 250 µM thick brain slices utilizing the third ventricle and retroflexes, as midbrain region landmarks (approximately A/P: −3.2 mm, M/L: ± 0.6 mm, D/V: −4.5 mm). Once located, the VTA was punched out using a blunt 16-gauge needle (I.D. 1.503 mm) and placed into RNeasy lysis buffer (Qiagen) and stored in a −80 °C freezer, after which mRNA extraction with RNeasy kit (Qiagen) was performed within 24 h. After mRNA extraction, reverse transcription was performed with iScript cDNA Synthesis Kit (BioRad), following the prescribed protocol, with a final reaction mixture of 50 μL. This mixture was then cycled in a C1000 Thermocycler (BioRad) under the following conditions: 25.0 °C for 5 min, 42.0 °C for 60 min, and 85 °C for 5 min. For qRT-PCR, all primers for tested targets were designed using Primer-BLAST software (NCBI), with identical parameters (T_m_, GC content, minimum primer length) for each primer set. The VTA cDNA libraries from each sample then underwent quantitative PCR in triplicate using an Opus quantitative PCR machine (BioRad) and SsoFast EvaGreen Supermix (BioRad). PCR for each sample to the followed this protocol: 98 °C hot start for two minutes, followed by 45 cycles of 98 °C for 2 s, 57 °C for 5 s (as per Bio-Rad instructions for SsoFast EvaGreen). Amplification was measured by increased relative fluorescence during each cycle and a cycle threshold (Ct) value was assigned to each target using BioRad CFX Manager software. All samples of each target were examined on the same plate to ensure accuracy of Ct values. Amplification specificity was confirmed via melt curve analysis. In our previous study, we verified proper amplification of each cellular target using 4% agarose gel electrophoresis to verify amplicon size (Ostlund et al. 2022).

### Statistical analyses

For analysis of the electrophysiological experiments, the peak amplitude of the electrically evoked EPSC was calculated using Clampfit 10.5 software (Molecular Devices) and graphed using Origin 7.5. Within individual experiments, current amplitudes were averaged by minute (6 sweeps/min). Cells were discarded if the access resistance changed by more than 15% during the experiment. Statistical analysis was performed in R, using a paired Student’s t-test to assess significant changes in EPSCs within the same treatment group. The t-tests are a comparison of the average values of the last 5 min of the baseline (pre HFS) to 15–20 min post-HFS. To assess differences in the normalized post-HFS EPSC amplitude between treatment groups, a 3-way ANOVA was used to determine if there was a significant interaction, with time as a within-subjects factor, and age and treatment as between-subjects factors. After a significant interaction was detected, a one-way ANOVA with a Tukey post-hoc analysis was used to assess differences in normalized post-HFS EPSC amplitude between groups. Alpha level was set to 0.05. Our previous work revealed no difference in this LTD between males and females (Ostlund et al. 2022); therefore, data from males and females were grouped together for all analyses.

For analysis of the qRT-PCR data, relative quantities of gene expression were determined using Microsoft Excel and the Livak and Schmittgen ΔΔCt/Cq method (Livak and Schmittgen 2001). Both 18S ribosomal and GAPDH were averaged to function as a transcription baseline (Riedel, et al. 2014). Statistical analysis was done at the level of ΔΔCt (Yuan et al. 2006). A 2-way ANOVA with treatment and age as between-subjects factors was used to determine significant alterations to mRNA expression, after which a Tukey post-hoc analysis was performed to determine which transcripts had been significantly altered. Data was compiled and presented as a ratio of 2-ΔΔCt normalized to vehicle-treated adult mice. Targets for PCR were selected to examine changes in various endocannabinoid elements (CB1, MAGL, FAAH, DALα), elements known to be involved in plasticity (GluA1, BDNF) and an epigenetic modifier (HDAC3).

## Results

### Three-day THC exposure eliminates LTD in young, but not adult mice

**Fig. 1:**
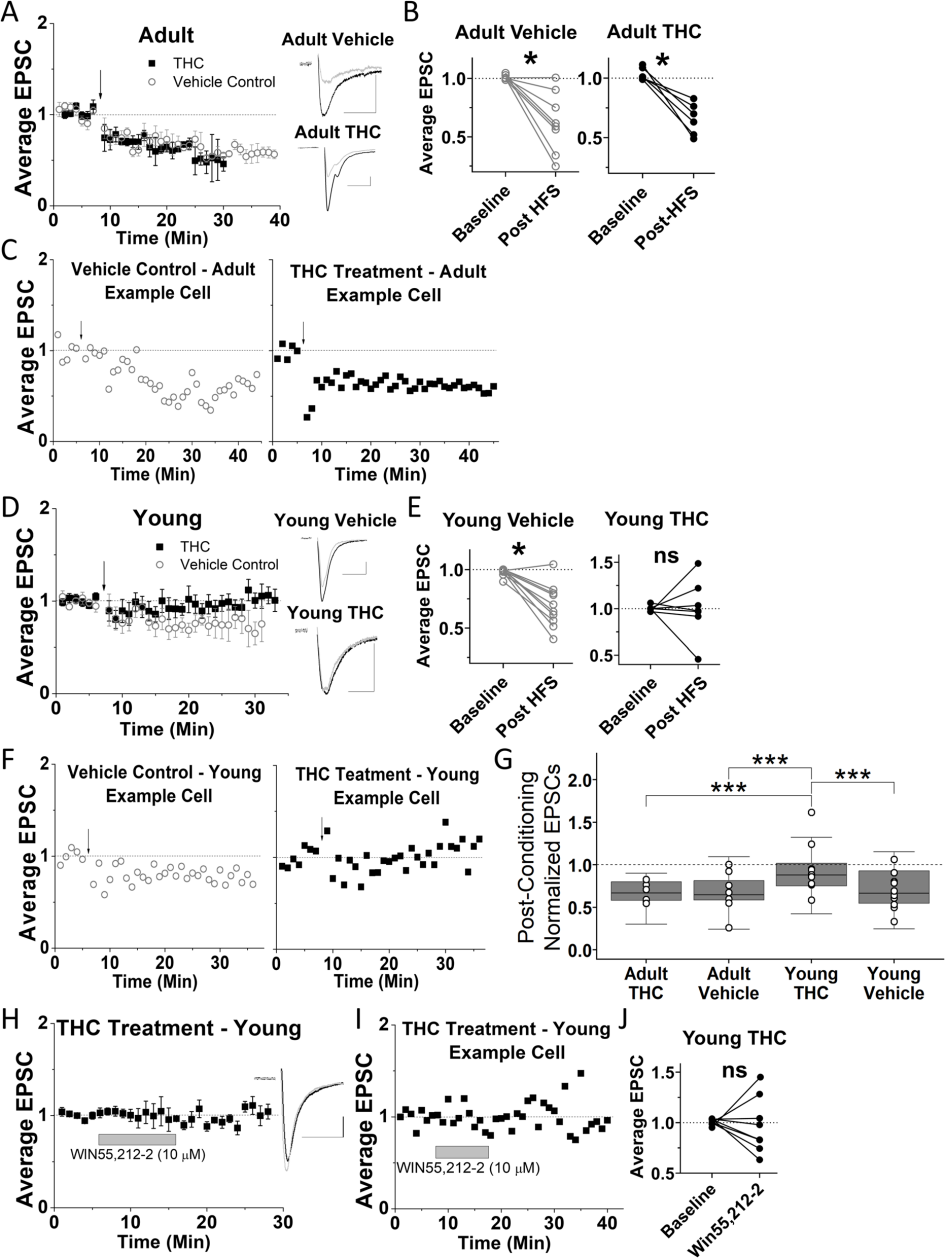
Three-day THC exposure eliminates long-term depression (LTD) in VTA GABA cells of young, but not adult mice. Glutamate-mediated excitatory postsynaptic currents (EPSCs) were recorded from GAD67-GFP-positive VTA GABA cells in brain slices. **A** Scatter plots showing averaged EPSCs before and after HFS (arrow) used to induce plasticity for adult mice treated with 3 treatments (1/day) of THC (10 mg/kg) or a vehicle control (EtOH (1µL/g) in saline). LTD is present in both cases. Insets in Fig. 1 are averaged EPSC traces (12 traces averaged) taken from before (black) and 13–15 min after HFS conditioning (gray). Scale bars are 50 pA, 10 ms. **B** Analysis using a paired student’s t-test shows that EPSC amplitude following HFS was significantly decreased compared to baseline for adult mice treated with a vehicle control (*n* = 8, *p* < 0.01, t = 4.08, df = 7) or THC (*n* = 6, *p* < 0.01, t = 5.67, df = 5). **C** Example experiment showing individual cells for both of the adult treatment groups. **D** LTD is eliminated in young mice treated with 3 treatments (1/day) with THC (10 mg/kg), while it remained in vehicle controls (EtOH (1µL/g) in saline). **E** Analysis using a paired student’s t-test shows that EPSC amplitude following HFS was significantly decreased compared to baseline for young mice treated with a vehicle control (*n* = 10, *p* < 0.001, t = 5.2, df = 9) but there was no significant difference between baseline and post-HFS EPSC amplitude for young mice treated with THC (*n* = 10, *p* = 0.99, t = 0.013, df = 9). **F** Example experiments showing individual cells for both young treatment groups. **G** Further analysis of normalized post-HFS EPSC amplitude employed a one-way ANOVA with a Tukey post-hoc analysis to compare between treatment groups. This analysis revealed that the post-HFS EPSC amplitude for adult mice treated with THC was not significantly different from adult mice treated with the vehicle control (*p* > 0.5, *F* = 0.014, df = 3, 151). However, young mice treated with THC showed significantly greater post-HFS EPSC amplitude compared to young mice treated with vehicle (*p* < 0.001, *F* = −0.23, df = 3, 151) and compared to adult mice treated with THC (*p* < 0.001, *F* = 0.27, df = 3, 151) or adult mice treated with a saline control (*p* < 0.001, *F* = 0.25, df = 3, 151). **H** There is a lack of WIN55,212–2-induced depression of glutamatergic currents in VTA GABA cells of young mice treated with 3-days of THC. **I** Example experiment demonstrate no effect of WIN55,212–2 in an individual cell of a young mouse treated with THC. **J** Analysis using a paired student’s t-test illustrates that EPSC amplitude following bath application of WIN55,212–2 was not significantly altered compared to baseline for young mice treated with 3 days of THC (*n* = 7, *p* = 0.95, t = 0.066, df = 6). Note: for panels B, E and I, t-tests are a comparison of the average baseline (5 min just before HFS or drug) and the average of 15–20 min post stimulation. All plots are normalized to the last 5 min of baseline and represent EPSC amplitude means with standard error of the mean (SEM). Arrow indicates 100-Hz HFS conditioning induction (4 stimuli, each for a period of 1 s, and each separated by 20 s). *, *p* < 0.05; ***, *p* < 0.001; ns, not significant

Our results reveal that 3-day THC exposure did not eliminate HFS-induced LTD in VTA GABA cells of adult mice, and EtOH vehicle-treated adult mice also continued to display LTD (Fig. 1A-C). Strikingly, LTD was eliminated in VTA GABA cells of young mice after the same THC treatment but was still present in the vehicle-treated young mice (Fig. 1D-F). These findings suggest that plasticity in young mice is more sensitive to THC-induced changes compared to adult mice, highlighting a novel age-dependent difference in neuroadaptations within the VTA. To assess differences in post-HFS EPSC amplitude between groups, a 3-way ANOVA was used, which revealed a significant effect of age (*p* < 0.05, *F*_1, 27_ = 7.319) but not treatment (*p* = 0.095, *F*_1,27_ = 2.99) or time (*p* = 0.91, *F*_2.9, 62_ = 0.17). The 3-way ANOVA also revealed a significant interaction between age and treatment (*p* < 0.05, *F*_1, 27_ = 4.697), but not between age, treatment and time (*p* = 0.38, *F*_4, 84_ = 1.07). To further explore the effects of age and treatment on post-HFS EPSC amplitude, a one-way ANOVA was used which revealed that the young mice treated with THC had significantly less plasticity (greater post-HFS EPSC amplitude) compared to all other groups (Fig. 1G). This one-way ANOVA also revealed that post-HFS EPSC amplitude for adult THC-treated mice was not significantly different from adult or young mice treated with vehicle injections. Next, to confirm the mechanism of 3-day THC elimination of LTD, we examined whether synaptic CB1 receptors remained functional at glutamatergic inputs using the specific CB1 agonist WIN55,212–2. After the 3-day THC-treatment, WIN55,212–2-induced depression was no longer present in young mice (Fig. 1H-J).

### Three-day THC treatment impacts mRNA expression in young and adult mice

**Fig. 2:**
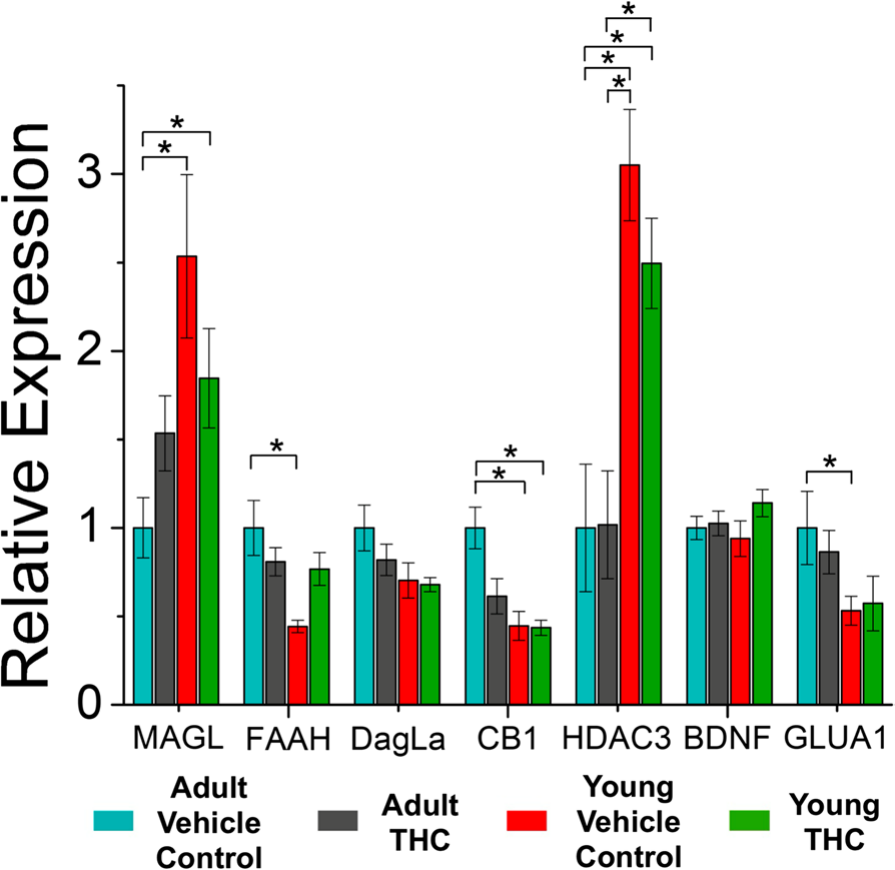
Age-dependent differences in VTA cell mRNA levels. THC-treated young mice express lower mRNA levels for CB1 when compared to vehicle control adult mice (*p* < 0.01, *F* = 1.19, df = 188). Vehicle-treated young mice also expressed lower mRNA levels for CB1 compared to vehicle-treated adults (*p* < 0.01, *F* = −1.16, df = 188). Young control mice express lower levels of fatty acid amide hydrolase (FAAH) mRNA than the adult control mice (*p* < 0.01, *F* = −1.17, df = 188). Adult control mice have lower mRNA expression levels of histone deacetylase 3 (HDAC3) compared to young control mice (*p* < 0.001, *F* = 1.61, df = 188) and young THC-treated mice (*p* < 0.001, *F* = −1.31, df = 188). Adult mice treated with THC also express lower levels of HDAC3 mRNA compared to young control mice (*p* < 0.001, *F* = −0.87, df = 188) and young THC-treated mice (*p* < 0.001, df = 188, *F* = 1.29). Adult control mice express lower mRNA levels of monoacylglycerol lipase (MAGL) compared to young control mice (*p* < 0.001, df = 188, *F* = 1.34) and compared to young THC-treated mice (*p* < 0.05, df = 188, *F* = −0.88). Young control mice also express lower levels of GluA1 mRNA compared to adult control mice (*p* < 0.05, df = 188, *F* = −0.93). Sample size was *n* = 10 for all groups except for the young vehicle treated mice, which has a sample size of 9. All samples are normalized to the mRNA level of the vehicle-treated adult mice. Hypothesis testing was performed with a 2-way ANOVA with treatment and age as between subject factors

Because our previous study identified changes to gene expression in young and adult mice after 7–10 days of THC-exposure, we sought to investigate whether age-related differences also occurred after only 3 days of THC-exposure. Differential mRNA expression revealed via qRT-PCR highlights some key differences between young and adult mice (Fig. 2). Namely, THC-treated and vehicle control young mice express lower levels of CB1 mRNA in the VTA compared to vehicle control adult mice. Additionally, there are higher levels of HDAC3 mRNA in the VTA of young THC-treated and young vehicle control mice compared to vehicle control and THC-treated adults, suggesting that differential rates of histone acetylation are age-dependent in the VTA. We also quantified age-dependent differences in mRNA levels of enzymes responsible for the metabolization of endocannabinoids. Specifically, MAGL mRNA expression was increased in control or THC-treated young mice compared to control adults, but mRNA levels for FAAH were decreased in control young mice compared to control adult mice. Levels of GluA1 mRNA were also decreased in young control mice compared to control adults, similar to what was seen in our previous study (Ostlund et al. 2022). However, although changes to mRNA levels were noted in young mice after 7–10 days of THC-treatment in our previous study, we did not identify any significant changes after 3-day THC exposure in adult or adolescent mice compared to their age-matched controls.

## Discussion

Alteration of VTA synaptic plasticity and changes to gene expression are hallmarks of drug-dependence research in animal models, as long-term neuroadaptations caused by drug exposure often correlate with drug-dependence (Koob and Volkow 2010; Kalamarides, et al. 2023). For example, findings in the nucleus accumbens (NAc) show that chronic THC-treatment eliminates glutamatergic LTD in NAc GABA neurons (Hoffman et al. 2003). Our previous studies demonstrated that glutamatergic LTD is also eliminated in VTA GABA cells of both young and adult mice after chronic THC treatment (Friend et al. 2017; Ostlund et al. 2022). Although, a single THC treatment eliminates endocannabinoid-mediated plasticity in the NAc and hippocampus (Mato et al. 2004), our previous data illustrate that a single THC treatment is not sufficient to eliminate endocannabinoid-mediated plasticity at VTA GABA cells (Friend et al. 2017). However, our current data illustrates that young mice undergo elimination of LTD after 3 days of THC treatment, but it is still present in adults after the same treatment. This short-term THC treatment is not commonly studied, as many of the synaptic effects of THC are seen after just one treatment (Mato et al. 2004; Good and Lupica 2010). However, 3-day treatment was ideal to further characterize the age-dependent acceleration of neuroadaptation in response to THC treatment in VTA GABA cells because LTD is not eliminated in young or adult mice after just one THC-treatment. A 3-day treatment was selected as a median timepoint to examine, based on 1- and 7-days exposure times previously examined where no differences were noted between age groups. The data reported here demonstrate that young mice are more sensitive to synaptic modifications caused by THC exposure than adults. This age-dependent differential response to THC-treatment highlights the nuanced ways in which age can influence the effect that drugs of abuse have on plasticity. Alterations to the surface expression or sensitivity of CB1 likely caused the elimination of plasticity, as the CB1 agonist WIN55,212–2 also failed to induce depression in young mice treated with THC for 3 days. Decreases in surface expression of presynaptic CB1 receptors after treatment with a CB1 agonist have been reported in other brain areas (Coutts et al. 2001). Elimination of WIN55,212–2-mediated depression was also noted in both young and adult mice after 7-10 days of THC-treatment previously (Friend et al. 2017; Ostlund et al. 2022).

Most drugs of abuse, including THC, cause epigenetic and transcriptional alterations that are thought to play a key role in dependence (Ostlund et al. 2022; Becker et al. 2017; Doyle and Mazei-Robison 2021; Zimmerman and Zimmerman 1990; Nannini et al. 2023; Miller et al. 2019). Furthermore, changes in gene transcription are often necessary for long-term synaptic plasticity to occur (McClung and Nestler 2008; Krug et al. 1984). In our previous study, we identified differences in mRNA levels within the VTA of young mice after THC treatment (Ostlund et al. 2022). That study examined heterogenous VTA brain punches, which we performed in this study to make both studies comparable (note that the previous study did not assess the impact of THC-treatment on adult mice). We identified that young mice express higher levels of histone deacetylase 3 (HDAC3) compared to adult mice, which was not noted in our previous study. HDAC3 activity promotes alterations in plasticity related to drug-seeking behaviors (Volkow et al. 2014). Upregulation of deacetylase in these young mice could result in a more closed chromatin structure, decreasing expression of regulatory genes that could have prevented malformation of VTA circuitry. Control or THC-treated young mice also express higher levels of monoacylglycerol lipase (MAGL) mRNA compared to adult control mice, mirroring expression patterns observed in the hippocampus (Piyanova et al. 2015), though this difference was not detected in our previous study. Because MAGL is a key enzyme in the hydrolysis of the endocannabinoid 2-AG, increased levels of MAGL could lead to decreased 2-AG signaling in the VTA of young mice. In contrast, young vehicle control mice express lower levels of fatty acid amide hydrolase (FAAH) mRNA compared to their adult counterparts. FAAH degrades anandamide, and thus lower FAAH expression could enhance anandamide levels in young mice.

Regarding 3-day THC impact on gene transcription, we assessed young and adult mice to determine whether transcriptional changes can occur after a shorter duration exposure to THC. While our previous study demonstrated increased mRNA levels for glutamate receptor 1 (GluA1) in young mice after 7–10 days of THC treatment (Ostlund et al. 2022), the 3-day THC-treatment did not affect GluA1 mRNA in young or adult mice. Three-day THC exposure also did not increase FAAH levels in either young or adult mice. However, our previous study identified that 7–10 days of THC exposure upregulates FAAH mRNA in young mice (Ostlund et al. 2022). This suggests that FAAH alterations are not involved in the elimination of endocannabinoid-mediated LTD in VTA GABA cells, as LTD was abolished following THC treatment despite no significant change to FAAH levels in young mice. Three days of THC treatment did not affect VTA mRNA levels of MAGL or diacylglycerol lipase (DAGL), similar to what was reported by others in the NAc after 3 days of THC exposure (Devuono 2018). However, in our previous study, 7–10 days of THC exposure did increase mRNA transcript levels of DAGL (Ostlund et al. 2022). Collectively, our data highlight differences between young and adult mice that may help explain the age-related disparity regarding this THC-induced elimination of plasticity after three-day THC exposure.

## Conclusions

Our data contribute to the expanding body of literature that underscores the ramifications of cannabis use in younger populations (Bara et al. 2021). The accelerated elimination of plasticity reported in young mice illustrates that susceptibility to cannabis-induced neuroadaptations can fluctuate throughout development. These findings highlight the need for further exploration into the age-related mesolimbic adaptations after cannabis exposure. Studying these age-related differences in reward system neurocircuitry is imperative to understanding any potential negative impact of either licit (prescribed) or illicit THC exposure throughout development.

## Data Availability

No datasets were generated or analysed during the current study.
